# Early health technology assessment of magnetic resonance-guided high intensity focused ultrasound ablation for the treatment of early-stage breast cancer

**DOI:** 10.1186/s40349-017-0101-3

**Published:** 2017-08-01

**Authors:** Floortje M. Knuttel, Sèvrin E. M. Huijsse, Talitha L. Feenstra, Chrit T. W. Moonen, Maurice A. A. J. van den Bosch, Erik Buskens, Marcel J. W. Greuter, Geertruida H. de Bock

**Affiliations:** 1 Department of Radiology, University Medical Center Utrecht, P.O. Box 85500, 3508 GA Utrecht, The Netherlands; 2 Department of Radiology, University Medical Center Groningen, University of Groningen, PO Box 30 001, 9700 RB Groningen, The Netherlands; 3 Department of Epidemiology, University Medical Center Groningen, University of Groningen, PO Box 30 001, 9700 RB Groningen, The Netherlands; 4 Center of Image Sciences, University Medical Center Utrecht, Utrecht, The Netherlands

**Keywords:** Breast cancer, MR-HIFU, HTA, Cost-effectiveness, Breast-conserving therapy

## Abstract

**Background:**

Magnetic resonance-guided high intensity focused ultrasound (MR-HIFU) ablation is in development for minimally invasive treatment of breast cancer. Cost-effectiveness has not been assessed yet. An early health technology assessment was performed to estimate costs of MR-HIFU ablation, compared to breast conserving treatment (BCT).

**Methods:**

An MR-HIFU treatment model using the dedicated MR-HIFU breast system (Sonalleve, Philips Healthcare) was developed. Input parameters (treatment steps and duration) were based on the analysis of questionnaire data from an expert panel. MR-HIFU experts assessed face validity of the model. Data collected by questionnaires were compared to published data of an MR-HIFU breast feasibility study. Treatment costs for tumours of 1 to 3 cm were calculated.

**Results:**

The model structure was considered of acceptable face validity by consulted experts, and questionnaire data and published data were comparable. Costs of MR-HIFU ablation were higher than BCT costs. MR-HIFU best-case scenario costs exceeded BCT costs with approximately €1000. Cooling times and breathing correction contributed most to treatment costs.

**Conclusions:**

MR-HIFU ablation is currently not a cost-effective alternative for BCT. MR-HIFU experience is limited, increasing uncertainty of estimations. The potential for cost-effectiveness increases if future research reduces treatment durations and might substantiate equal or improved results.

## Background

Breast cancer is the most common malignancy in women worldwide and its incidence is increasing [1, 2]. As a result of national screening programmes, most breast cancers are detected at an early stage [3]. Early stage breast cancer is usually treated with breast conserving therapy (BCT), which consists of lumpectomy combined with radiotherapy, followed by systemic therapy in patients deemed at high risk of metastases [4]. The overall prognosis after BCT is good, i.e. survival is similar to more radical mastectomy [5]. However, any surgical treatment always bears a risk of impaired cosmetic results and complications [6–8].

Currently, a shift towards non-surgical and less invasive treatment has been observed in several clinical trials, assessing the feasibility and efficacy of minimally invasive therapies [9–12]. One of these novel treatments is Magnetic Resonance guided High Intensity Focused Ultrasound ablation (MR-HIFU) [13]. Using focused ultrasound beams with a high power MRI-integrated HIFU systems heat breast tumours to high temperatures, inducing coagulation necrosis. Possible advantages of MR-HIFU ablation are a lower risk of complications such as infection and haemorrhage, improved cosmetic outcome and the possibility to offer the treatment in an outpatient setting without general anaesthesia. MRI-guidance is used for tumour visualization and temperature measurement during the procedures [13–15]. Initial clinical MR-HIFU studies report the treatment of approximately 122 malignant breast tumours, of which 77 were excised afterwards to assess histopathological response. The percentage of complete tumour ablation in these small feasibility studies varies from 16.7 to 90% [16–25].

Besides effectiveness, potential cost-effectiveness is a relevant aspect of introducing a new technique. MR-HIFU ablation will only have the potential to become a primary treatment in the future if its cost-effectiveness is acceptable compared to surgical treatment. Because its costs have not been assessed yet, the purpose of the current study was early health technology assessment. While assuming equal effectiveness of MR-HIFU and BCT, costs for treatment using MR-HIFU ablation compared to BCT were estimated. Additionally, the influence of several treatment-related features on these costs was assessed.

## Methods

A decision tree model was developed to evaluate the additional costs of MR-HIFU ablation as a replacement of BCT for the treatment of early-stage breast cancer and to what extent these costs are influenced by several treatment-related features. Equal effectiveness of MR-HIFU ablation and BCT was assumed for these analyses, because of the limited amount of clinical data of MR-HIFU treatments [26, 27]. Model input data were collected in a systematic way. Where possible, parameter estimates were based on literature. For parameters that were not available in literature, a survey among experts was performed. MR-HIFU experts were asked to assess the validity of the model. In addition, the model input was validated by comparison of treatment duration estimates to a recent publication on the feasibility and safety of MR-HIFU ablation [28].

### BCT and MR-HIFU scenarios

BCT was compared with MR-HIFU ablation. For BCT, treatment consisted of surgery with sentinel lymph node procedure, hospital admission, histopathological examination of excision specimen and adjuvant treatment in most cases. MR-HIFU treatment comprised a pre-treatment MRI scan, separate pre-treatment sentinel lymph node procedure, MR-HIFU ablation in day care setting and adjuvant treatment in most cases. Follow-up was not taken into account in this early health technology assessment (HTA).

### Patients

MR-HIFU ablation was considered most suitable for patients with early stage breast cancer with a maximum diameter of three centimetres with no malignant foci at a larger distance than 1 cm from the tumour edge [29]. Additional exclusion criteria for MR-HIFU ablation were: ductal carcinoma in situ (DCIS) and lobular histological type, as both increased the risk of incomplete resection and ablation [30, 31], the presence of axillary lymph node metastases and all contra-indications for MRI. Furthermore, patients could be excluded from MR-HIFU treatment due to the following factors assessed on pre-treatment MRI: tumour not reachable for the ultrasound beam, or distance from tumour to skin or pectoral muscle < 1 cm [28].

### Model

**Fig. 1:**
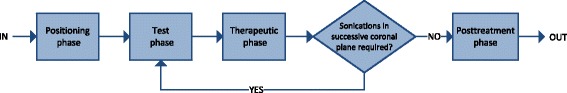
Overview of the four phases of MR-HIFU treatment

A model comprising the MR-HIFU treatment as performed with the dedicated MR-HIFU breast system (Sonalleve-based prototype, Philips Healthcare, Vantaa, Finland) was developed [32]. The model distinguished four separate phases: patient positioning on the MR-HIFU system, test phase (establishment of the exact treatment focus and treatment planning), therapeutic phase (the actual tumour ablation) and post-treatment phase (Fig. 1).

In the positioning phase, the patient was positioned on the HIFU table, which was then placed in the MR scanner, and tumour reachability for the HIFU beams was checked. Next, sedation analgesia was administered and target definition was performed based on MR images. In the test phase the respiratory breathing pattern was tracked to correct for breathing artefacts during proton resonance frequency shift (PRFS) thermometry [15]. Additionally, in the test phase the exact location of the focal point was checked and adjusted if necessary. In the therapeutic phase sonications with a higher power than used in the test phase were applied. Using PRFS thermometry maps, the temperature rise in the targeted tissue was followed to ensure that ablative temperatures were reached. Test sonications were applied again after switching to the next treatment slice. In the post-treatment phase contrast-enhanced MRI was performed to evaluate treatment results and sedation analgesia was ceased. The patient was admitted to a clinical ward for observation during the next four hours. Modelling was performed in MATLAB (R2014a). The conceptual model was tested for face validity with MR-HIFU experts involved in the MR-HIFU ablation feasibility and safety study performed with the aforementioned HIFU breast system [28].

### Model input data

For an overview of the model input parameters, see Table 1 and Table 2. To estimate the yearly number of early stage breast cancer (stage I and stage II tumours (≤3 cm in diameter)) patients, data from the Netherlands Comprehensive Cancer Organisation were used [33]. Several parameters concerned duration of treatment steps and probability of events related to treatment, e.g. repositioning a patient. These were based on a questionnaire filled in a by a team of (inter) national experts (physicians involved in breast cancer treatment, physicists, technicians and physicians with MR-HIFU experience). Durations were estimated by seven experts, probabilities by four of those seven experts.

**Table 1:** Duration of treatment steps and probability of events for clinically applied MR-HIFU breast cancer treatments as predicted by experts compared to data from the MR-HIFU feasibility study

Treatment phase	Parameter	Experts				Faesibility study [28]
		Unit	Median	Min	Max	Median
Positioning	Time patient verification	min	15	10	25	11.5
	Time verification reachability	min	15	7	20	14.5
	Time target definition	min	8	5	15	n/a
	Chance of repositioning	-	0.30	0.10	0.75	n/a
Test	Time to place navigator	min	2	1	10	1
	Time MRI scan	min	2.5	1	15	2.5
	Time for treatment planning	min	2	1	5	n/a
	Time to fill LUT	min	2.75	0	5	n/a
	Test sonication and check focal point	min	3	1	5	4
	Chance of adjustment focal point per coronal plane	-	0.55	0.20	0.90	n/a
Therapeutic	Time therapeutic phase	min	0.50	0.25	0.75	n/a
	Cooling time after each sonication	min	3.5	1	10	n/a
	Chance of abortion per coronal plane	-	0,10	0,05	0,20	n/a
	Chance of resonication per coronal plane	-	0.20	0.10	0.30	n/a
Post-treatment	Time clinical ward	min	240	120	300	n/a
	Chance of complications	-	0.015	0.01	0.03	n/a

*n/a* not available

**Table 2:** Resource items for diagnosis and treatment unit prices for the Netherlands, and sources

Cost category	Unit Price	Source
Diagnostics		
Pathology and evaluation	€ 83	Flobbe et al (2004)
DBC sentinel node procedure	€ 367.71	Passantenprijslijst UMC Utrecht - Overige zorgproducten, 2014
Treatment items		
Estimated costs for using MR scanner and HIFU device	€ 11/min	Overzicht tarieven onderlinge dienstverlening UMCG; 2016
Anesthetics costs	€ 2.44 /min	Division of Vital Functions, UMC Utrecht, 2015
Costs clininal ward	€ 0.44/min	CVZ, Handleiding voor kostenonderzoek, 2010;
Costs technician	€ 0.42/min	CVZ, Handleiding voor kostenonderzoek, 2010; CAO universitair medische centra 2015–2017
Costs nurse	€ 0.42/min	CVZ, Handleiding voor kostenonderzoek, 2010; CAO universitair medische centra 2015–2017
Costs radiologist	€ 1.33/min	CVZ, Handleiding voor kostenonderzoek, 2010; CAO universitair medische centra 2015–2017
DBC DCE MRI	€ 331	Dutch Healthcare Authority (NZA) 2012
Breast conserving therapy	€ 1109	https://www.zorginstituutnederland.nl/
Radiation therapy	€ 3179	Flobbe et al (2004); LPRM and NABON (2000); Slotman et al (2000)
Chemo therapy	€ 1044	Flobbe et al (2004); Slotman et al (2000)
Hormonal therapy	€ 806	Flobbe et al (2004); Slotman et al (2000)
Specialist Visits	€ 738	Flobbe et al (2004);
Specialist visits (adjuvant therapy)	€ 1966	Flobbe et al (2004);
Hospital stay	€ 1753	Flobbe et al (2004); Oostenbrink et al (2000)

To estimate the time that was needed for MR-HIFU treatment, a simplified tumour model was assumed with sphere-shaped tumours with a diameter of 1, 2 or 3 cm. A safety margin of 0.5 cm was added to the tumour size, resulting in spheres with a diameter of 2, 3 or 4 cm. The treatment cells of the HIFU device were considered cylindrically shaped with a diameter of 3, 6, 9 or 12 mm in the coronal plane and a height of 2, 4, 6 or 8 in the sagittal plane respectively. These are the approximate values provided by the dedicated MR-HIFU system [28, 32]. The number of sonications required for tumours of different diameters was approximated by assuming cylindrical shaped treatment cells covering a sphere shaped tumour. The number of sonications varied with the height and diameter of treatment cells, as follows: treatment cells with a diameter of 9 mm and height of 6 mm resulted in 17 sonications for tumours of 1 cm, 50 sonications for tumours of 2 cm and 110 sonications for tumours of 3 cm. Treatment cells with a diameter of 12 mm and height of 8 mm resulted in 9, 25, and 50 sonications respectively.

### Cost data

The average costs of BCT of the aforementioned patient population were based on a database comprising 1,345 breast cancer patients [34]. Hereby a weighting to the amount of women undergoing lumpectomy with or without adjuvant therapy, i.e. systemic therapy (hormonal therapy and chemotherapy) and radiotherapy, was done [35–37]. To estimate the costs for using the MR scanner and HIFU device, tariffs for MR procedures that were comparable in complexity, such as brain and heart MR imaging were used as a proxy [38]. Costs for the sentinel node procedure and contrast enhanced MRI were based on their tariffs [39]. Depreciation and maintenance costs of devices were incorporated in these tariffs. Costs of the additional MR-HIFU treatment components, e.g. sedation costs, were based on hospital specific rates. Costs of personnel present during the procedures was based on estimates of time needed multiplied by hourly costs, based on the guidance of the National Health Care Institute (Dutch: *Zorginstituut Nederland*) [40]. Costs of follow-up were not taken into account. Costs were indexed to 2014 by using consumer price index numbers [41].

### Analysis

The costs of MR-HIFU ablation were based on the MR-HIFU submodel. The lowest and median estimates of time needed obtained with expert questionnaires were used to calculate the most optimistic (‘best case’) and less optimistic (‘median case’) MR-HIFU treatment scenarios. MR-HIFU treatment costs for tumour sizes of 1, 2 and 3 cm were calculated. This was done for treatment cells of 9 × 6 mm and 12 × 8 mm. These costs were compared to the average BCT costs. Tornado diagrams were constructed to describe the sensitivity of costs to parameter estimates.

## Results

### Patients

Taking all possible exclusion criteria for MR-HIFU treatment into account, the proportion of patients eligible for MR-HIFU treatment was 11.9% of all patients diagnosed with breast cancer (Table 3) [31, 42–52].

**Table 3:** Proportion of patient eligible for MR-HIFU treatment

Inclusion criteria	Proportion (%)
Tumour ≤ 3 cm	78.6
No lymph node metastasis	65.0
No lobular subtype	90.2
No EIC	84.6
No previous surgery	91.3
No renal insufficiency	97.3
Not multifocal	82.0
No BRCA mutation	97.4
Tumour reachable	66.3
Distance to skin ≥ 1 cm	65.0
Eligible patients^a^	11.9

*EIC* extensive intraductal component

^a^i.e. all of the inclusion criteria present

### Model

The structure of the model was considered of acceptable face validity by experts consulted. Model input parameters on analogous variables derived from actually observed data were comparable to the answers obtained through the questionnaires [28]. The duration and chances of occurrence of the different MR-HIFU treatment steps, compared to the data observed in the feasibility study, is shown in Table 1.

### Costs of MR-HIFU ablation and BCT

The costs of MR-HIFU ablation for best and median case scenarios and two different cell sizes and the costs of BCT for tumours of 1, 2 or 3 cm are displayed in Table 4. The larger the treatment cell, the lower the MR-HIFU costs and the shorter the procedure time. For all variants, the costs of MR-HIFU ablation were higher than the costs of BCT. When using treatment cells size of 12 × 8 mm, the best case scenario costs of MR-HIFU ablation approached those of BCT.

**Table 4:** Costs of MR-HIFU ablation for best and median case scenarios compared to costs of BCT

	MR-HIFU ablation								BCT
	Median case				Best case				
	Treatment cell size (mm)				Treatment cell size (mm)				
	6 × 9		12 × 8		6 × 9		12 × 8		
Tumour size (mm)	Costs (€1000)	Time (h)	Costs (€1000)	Time (h)	Costs (€1000)	Time (h)	Costs (€1000)	Time (h)	Costs (€1000)
10	11.5	4.4	10.0	2.8	8.5	1.1	8.2	0.8	7.1
20	15.5	8.8	12.5	5.4	9.1	1.9	8.6	1.3	8.1
30	23.3	17.4	15.5	8.8	10.5	3.4	9.1	1.9	8.1

### Factors influencing treatment costs

**Fig. 2:**
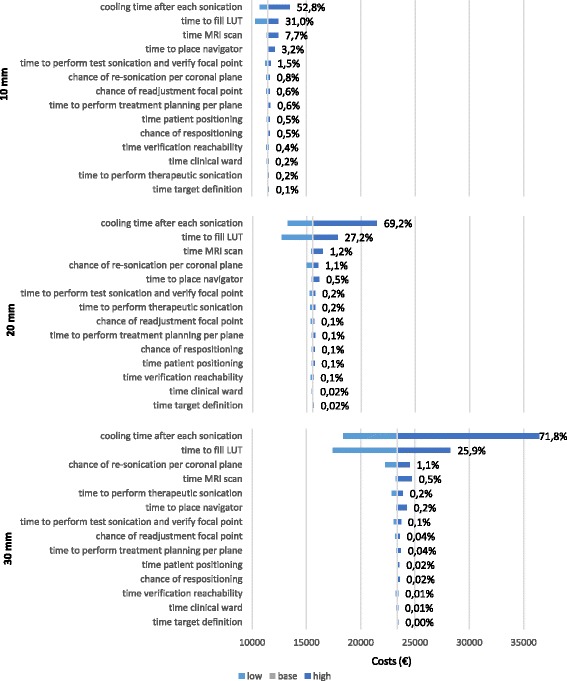
Tornado diagrams presenting sensitivity to parameter values of the difference in treatment costs for tumours of 10, 20 and 30 mm with an added safety margin of 5 mm assuming treatment cells of 9 × 6 mm

**Fig. 3:**
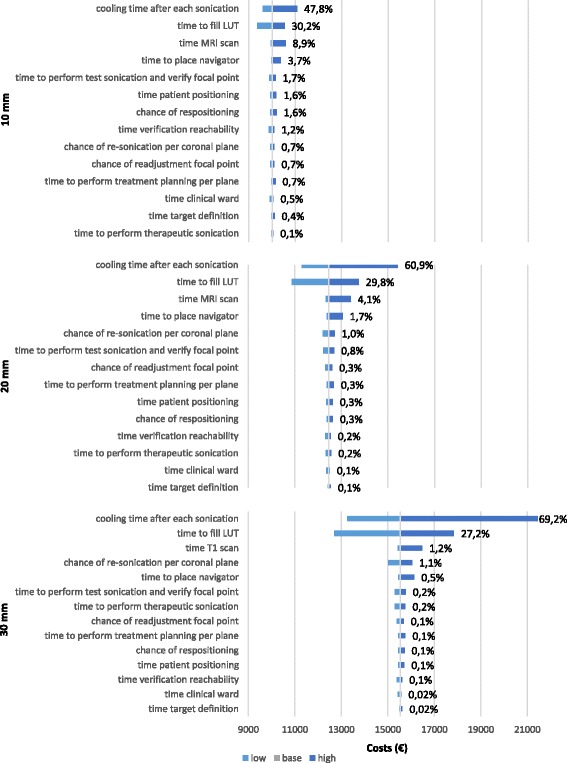
Tornado diagrams presenting sensitivity to parameter values of the difference in treatment costs for tumours of 10, 20 and 30 mm with an added safety margin of 5 mm assuming treatment cells of 12 × 8 mm

Factors contributing most to the total treatment costs were: cooling time after each sonication, and time required for breathing correction. For 9 × 6 mm treatment cells the average sensitivity for cooling time over all tumour sizes was 64.6 ± 10.3%, for the breathing correction this was 28 ± 2.7%. For treatment cells of 12 × 8 mm this was 59.3 ± 10.8% and 29.1 ± 1.6% respectively. Changes in these two parameters had larger impact on the cost estimations of treatments with smaller treatment cells than with larger treatment cells. The sensitivity of each model input parameter on model output is shown in Table 5 in tornado diagrams. The sensitivity values for both 9 × 6 mm and 12 × 8 mm treatment cells are presented in respectively Fig. 2 and 3.

**Table 5:** Variance (%) of uncertainties in tornado diagrams. A safety margin of 0.5 cm is added to the tumour size

Treatment phase	Tumour size (mm)^a^	10		20		30					
	Treatment cell size (mm)	9×6	12×8	9×6	12×8	9×6	12×8	9×6		12×8	
	Parameter							Mean	sd	Mean	sd
Positioning	Time patient positioning	0.5	1.6	0.1	0.3	0.0	0.1	0.2	0.3	0.7	0.8
	Time verification reachability	0.4	1.2	0.1	0.2	0.0	0.1	0.2	0.2	0.5	0.6
	Time target definition	0.1	0.4	0.0	0.1	0.0	0.0	0.0	0.1	0.2	0.2
	Chance of repositioning	0.5	1.6	0.1	0.3	0.0	0.1	0.2	0.3	0.7	0.8
Test	Time MR scan	7.7	8.9	1.2	4.1	0.5	1.2	3.1	4.0	4.7	3.9
	Time to place navigator	3.2	3.7	0.5	1.7	0.2	0.5	1.3	1.7	2.0	1.6
	Time to perform treatment planning per coronal plane	0.6	0.7	0.1	0.3	0.0	0.1	0.2	0.3	0.4	0.3
	Time to fill LUT	31.0	30.2	27.2	29.8	25.9	27.2	28.0	2.7	29.1	1.6
	Time to perform test sonications and verify focal point	1.5	1.7	0.2	0.8	0.1	0.2	0.6	0.8	0.9	0.8
	Chance of readjustment focal point	0.6	0.7	0.1	0.3	0.0	0.1	0.2	0.3	0.4	0.3
Therapeutic	Time to perform therapeutic sonication	0.2	0.1	0.2	0.2	0.2	0.2	0.2	0.0	0.2	0.1
	Cooling time after each sonication	52.8	47.8	69.2	60.9	71.8	69.2	64.6	10.3	59.3	10.8
	Chance of resonication per coronal plane	0.8	0.7	1.1	1.0	1.1	1.1	1.0	0.2	0.9	0.2
Post treatment	Time clinical ward	0.2	0.5	0.0	0.1	0.0	0.0	0.1	0.1	0.2	0.3

Sensitivity (variance) was calculated by calculation of the swing square relatively to the total swing square. Hereby, the swing is the range of cost values for a given uncertainty

^a^A safety margin of 0.5 cm was added to the tumour size

## Discussion

To our knowledge, this is the first study on the potential cost-effectiveness of MR-HIFU ablation of breast cancer. This early health technology assessment suggests that MR-HIFU ablation was more expensive than BCT. When larger treatment cells were assumed, the potential for MR-HIFU ablation to have comparable costs increased. The duration of certain treatment steps including cooling time after each sonication and the time needed to apply breathing correction, had most impact on MR-HIFU costs. Importantly, the analyses were performed under the assumption that MR-HIFU ablation and BCT are equally effective.

Due to the limited amount of MR-HIFU treatment data, the clinical effectiveness, complication rate and the effect on quality of life and cosmetic outcome is still scarce. Therefore, the effectiveness of MR-HIFU ablation was considered equal to BCT and complications were not taken into account. However, if MR-HIFU treatment would be optimized and surgical excision would be omitted in the future, surgical complications and breast deformation might occur less frequently. This is expected to have a favourable effect on quality of life and cosmetic outcome. Even if MR-HIFU ablation would be slightly less effective than BCT, some patients might still prefer MR-HIFU because of its favourable effect on cosmetic outcome or reduced risk of complications. Especially elderly patients with more comorbidities and shorter life expectancy may be interested in MR-HIFU ablation. Quality of life measures are usually incorporated in cost-effectiveness analyses as well, and hence better treatment associated utility scores would increase the potential for MR-HIFU to become cost-effective [53].

Our results indicate that in order to improve the cost-effectiveness of MR-HIFU ablation treatment time should be reduced. The currently used cooling times are applied to guarantee safety. If more clinical experience with MR-HIFU ablation is gained, shorter cooling times may possibly appear equally safe. Being able to measure the temperature in the surrounding (adipose) tissue in the breast may contribute by providing real time temperature measurements during cooling. Possible methods for thermometry in adipose tissue are T2-weighted thermometry [54, 55] or a hybrid method for thermometry in fat and adipose tissue at the same time [56, 57]. Implementation of these techniques will become possible in the near future. The time needed for breathing correction could be improved by using sedation that results in a more regular breathing pattern, or even obviates the need for breathing correction. Furthermore, the parameters of breathing correction could be made more flexible enabling sonications to start after a shorter period. To what extent this is possible should be clinically investigated first and is not expected in the near future. Another reason for the higher costs of MR-HIFU ablation is the separately performed sentinel lymph node procedure, which is incorporated in the operation for BCT.

Strong points of this study are that it provided the first data on the potential cost-effectiveness of MR-HIFU ablation and that MR-HIFU experts validated the applied treatment models. Due to the lack of empirical treatment data, other sources for model input were needed. Estimations of experts are the most accurate option in this case. Furthermore, the duration of several treatment elements was validated with the published MR-HIFU breast study in which the dedicated MR-HIFU breast system was used [28].

Some limitations of the present study should also be acknowledged. First, MR-HIFU treatment duration may have been overestimated as a result of the lack of experience. As mentioned before, cooling times may be unnecessarily prolonged. Besides, no experience with total tumour ablation exists yet, possibly affecting some estimations. Second, treatment cells were assumed to be cylindrical shaped to enable calculations of the number of sonications required per tumour. The MR- HIFU breast system provides treatment cells with the shape of an oblate ellipse. This difference in shape may have affected the results. Third, the costs of BCT may have been overestimated, as this cost estimate also comprised patients with lobular carcinoma and positive axillary lymph nodes. However, patients with a tumour of the lobular subtype are considered ineligible for MR-HIFU treatment, as lobular breast cancer has a higher risk of incomplete resection and is consequently more expensive to treat. The same applies for patients with positive axillary lymph nodes. Fourth, our estimations were based on the dedicated MR-HIFU breast system used in our centre (Sonalleve-based prototype, Philips Healthcare, Vantaa, Finland). Other MR-HIFU systems exist and it is unclear if our results would be generalizable to these systems. Fifth, the cost data is based on the local health system. This means that the cost data cannot be extrapolated to other countries. However, we have generated a model that is usable internationally. Lastly, in the performed early HTA indirect costs were not taken into account. For example, the expected duration of absence at work will be longer after breast surgery than after MR-HIFU. As a result, the costs of BCT may have been underestimated in this study.

Several issues need to be addressed before MR-HIFU ablation can become a clinical treatment. First of all, the clinical efficacy of MR-HIFU has to be proven. MR-HIFU is competing with a very reliable and well-established treatment option, BCT. Clinical trials on MR-HIFU are therefore hard to perform and require many participants. Other concerns are that no surgical excision specimen is available after MR-HIFU treatment, the indication for adjuvant treatment needs to be assessed prior to treatment. Furthermore, margin status cannot be assessed, which may be resolved by frequent follow-up with MRI. In conclusion, MR-HFIU ablation is a novel treatment which is currently not ready for clinical implementation, but recent developments are promising for the future.

## Conclusions

We tentatively conclude that MR-HIFU ablation currently is not a cost-effective alternative to BCT. The costs of MR-HIFU ablation are mostly affected by the long duration of certain treatment components, i.e. cooling time after sonications and the time needed to apply breathing correction. Furthermore, costs were influenced by the size of treatment cell used and decreased with larger treatment cell size. Being an early HTA analysis, the study had to be based on several assumptions and estimations, because the experience of MR-HIFU ablation is still quite limited. Therefore, our results may give important directions for future development of MR-HIFU ablation. Especially cooling time in between sonications and accurate breathing correction take relatively long and thus appear relevant targets for further innovation.

## Abbreviations

BCT: Breast conserving treatment
DCIS: Ductal carcinoma in situ
HTA: Health technology assessment
MR-HIFU: Magnetic resonance guided high intensity focused ultrasound ablation
PRFS: Proton resonance frequency shift
